# Pregnancy risk in beef and dairy cows after supplementing semen with transforming growth factor beta-1 at the time of artificial insemination

**DOI:** 10.1093/jas/skae169

**Published:** 2024-06-27

**Authors:** Katelyn L M Fritsche, Jason K Ahola, Pablo J Pinedo, George E Seidel, Ryan D Rhoades, Jeffrey S Stevenson, K C Olson, John R Jaeger, David M Grieger, John J Bromfield

**Affiliations:** Department of Animal Sciences, Colorado State University, Fort Collins, CO 80523, USA; Department of Animal Sciences, Colorado State University, Fort Collins, CO 80523, USA; Department of Animal Sciences, Colorado State University, Fort Collins, CO 80523, USA; Animal Reproduction and Biotechnology Laboratory, Colorado State University, Fort Collins, CO 80523, USA; Department of Animal Sciences, Colorado State University, Fort Collins, CO 80523, USA; Department of Animal Sciences and Industry, Kansas State University, Manhattan, KS 66506, USA; Department of Animal Sciences and Industry, Kansas State University, Manhattan, KS 66506, USA; Department of Animal Sciences and Industry, Kansas State University, Manhattan, KS 66506, USA; Department of Animal Sciences and Industry, Kansas State University, Manhattan, KS 66506, USA; Department of Animal Sciences, University of Florida, Gainesville, FL 32611, USA

**Keywords:** artificial insemination, beef cows, organic dairy cows, seminal plasma, transforming growth factor beta-1

## Abstract

Our objective was to determine if the addition of a concentrated human recombinant transforming growth factor beta-1 (**TGF**) to bovine semen at the time of AI would result in increased risk of pregnancy in beef and dairy cows. Suckled beef cows (*n* = 1,132) in 11 herds across 2 states and lactating dairy cows (*n* = 2,208) in one organic-certified herd were enrolled. Beef cows received fixed-time AI (**FTAI**) following a 7 d CO-Synch + controlled internal drug release estrous synchronization protocol. Dairy cows were inseminated following observation of natural estrus expression. Cows received either no treatment as a control (**CON**) or 10 ng of TGF in 10 μL added through the cut-end of a thawed straw of semen immediately prior to AI. At the time of FTAI of beef cows, the mean ± SD age was 5.0 ± 2.4 yr, BCS was 5.3 ± 0.7, and days postpartum was 78.2 ± 15.5 d. The overall pregnancy risk (PR) in beef cows was 55.2% to AI and 90.5% season-long. PR in beef cows was not affected (*P* = 0.27) by the addition of TGF (53.1% vs. 58.1%). Furthermore, there was no difference (*P* = 0.88) for season-long PR in beef cows that received TGF (91.2% vs. 91.5%). At the time of insemination of dairy cows, the mean ± SD lactation was 3.0 ± 1.3 lactations, BCS was 2.9 ± 0.3, days in milk was 115.6 ± 56.6 d, and cows had received 2.4 ± 1.5 inseminations/cow. The overall pregnancy risk to AI in dairy cows was 23.1%. PR to AI for dairy cows was not affected (*P* = 0.32) by addition of TGF (22.0% vs. 23.8%). In conclusion, PR to AI was not affected by addition of TGF to thawed semen immediately prior to AI in beef or dairy cows.

## Teaser

An attempt to improve pregnancy rate by adding back transforming growth factor beta-1 to semen used for artificial insemination. Supplementing transforming growth factor beta-1 did not improve the pregnancy rate in beef or dairy cattle.

## Introduction

Seminal plasma is known as both a transport and survival medium for mammalian sperm during and after ejaculation. It has been recognized for its active roles in targeting female reproductive tissues post-coitus and the potential for inducing modulations in the uterine environment (Robertson, 2005; Schuberth et al., 2008). It is thought that an inflammatory response following coitus in the female reproductive tract helps clear microorganisms and sperm cells from the tract (Hansen et al., 1987; Alghamdi et al., 2009). However, it has been proposed that seminal plasma-induced inflammation could aid in the establishment and maintenance of pregnancy by modifying the uterine microenvironment (Robertson, 2005; Bromfield, 2016). Seminal plasma-derived transforming growth factor beta-1 (**TGF**) has been identified as a major contributor to postcoital inflammation in the female reproductive tissues of both mice and humans (Tremellen et al., 1998; Robertson, 2005). Total TGF content of bull semen has been calculated at 63.4 ± 15.6 ng/ejaculate (Rizo et al., 2019). Postcoital inflammation is not well documented in cattle except for an influx of neutrophils (Howe and Black, 1963; Mattner, 1968), and more recently by changes in endometrial gene expression (Ibrahim et al., 2019; Rizo et al., 2019). Moreover, the supplementation of pooled seminal plasma at the time of AI in dairy cows increases heifer calf birth weight (Ortiz et al., 2019).

Cows that undergo AI with cryopreserved semen lack the seminal plasma concentrations normally found following natural service. Yet the relative success of pregnancy to AI with cryopreserved semen suggests that pregnancy can be routinely initiated in the absence of seminal plasma (Lima et al., 2009). Odhiambo et al. (2009) reported no effect on pregnancy rates in dairy cows (*n* = 800) that received 0.5 mL of seminal plasma or TGF (40 ng) at the time of AI versus control (37.8%, 36.3%, and 33.2%, respectively). Odhiambo et al. (2009) also reported no effect on pregnancy rates in beef cows (*n* = 763) that received 0.5 mL of seminal plasma or TGF (40 ng) at the time of AI vs. control (58.8%, 51.0%, and 55.1%, respectively). These studies did not observe an effect of either seminal plasma or TGF treatment in dairy or beef cows, likely due to the limited sample size.

We hypothesized that the addition of TGF to semen at the time of AI would increase the risk of pregnancy in cattle. Therefore, the objective of the current study was to determine if the addition of a concentrated human recombinant TGF to the cut-end of a thawed straw of semen at the time of AI affected the risk of pregnancy in lactating beef cows and organic dairy cows.

## Materials and Methods

The Colorado State University Animal Care and Use Committee approved all procedures conducted in this experiment.

The study was designed as a complete randomized experiment where both beef and dairy cows were assigned to receive AI with either control semen (**CON**) alone, or semen treated with 10 ng of human recombinant TGF (R & D Systems, Minneapolis, MN; #240-B). Beef cows at six locations (herds two through seven) were stratified by parity and then randomly assigned to either CON or TGF within stratification. Beef cows at five locations (herds 1 and 8 through 11) were assigned to either CON or TGF based on an even or odd ear tag number. Dairy cows were randomly assigned (every other animal) to receive either CON or TGF at the time of any AI. The study was completed during spring and summer.

The human recombinant TGF treatment contained 1 μg/mL TGFβ-1 in PBS, with 25 μg/mL of gentamicin and 50 μg/mL of bovine serum albumin. The TGF treatment was stored in 110 μL aliquots, enough for approximately 10 inseminations, and frozen at −80 °C. Prior to use the stored tube containing TGF was thawed by submerging a portion of the tube in a water bath thaw unit (~95.0 °C) utilized for thawing straws of semen. Once thawed, TGF was pipetted utilizing a 10 μL pipette and a disposable pipette tip to administer 10 μL of TGF (total of 10 ng of TGF) into the cut end of a thawed straw of conventional semen. Certified AI technicians then completed the insemination of each cow.

### Management of beef cows

Lactating spring-calving beef cows (*n* = 1,132) were enrolled and managed in 11 herds at 6 locations in two states (CO and KS) and were primarily Angus-based. Beef cows were managed on native pastures prior to and throughout the breeding season. All beef cows were subjected to the 7 d CO-Synch + controlled internal drug release (**CIDR**) estrous synchronization protocol. This included an injection of 100 mg gonadotropin-releasing hormone (**GnRH**; 2 mL Factrel i.m.; Zoetis, Florham Park, NJ) and a CIDR (1.38 g progesterone impregnated intravaginal insert; Zoetis) on day −7, CIDR removal and an injection of 25 mg prostaglandin F_2α_ (**PGF**; 2 mL Lutalyse HighCon i.m.; Zoetis) on day 0 concurrent with application of estrus-detection patches (Estrotect, Spring Valley, WI) to the tailheads in accordance with the manufacturer’s recommendation. Patch status was observed and recorded 66 ± 2 h post-PGF, at the time of fixed-time AI (**FTAI**), and estrus was determined to have occurred if > 50% of the patch had been activated. Cows with non-activated patches at FTAI received GnRH, while cows with activated patches at FTAI received no additional injections. Body condition score (1 = thin, 9 = obese; Bellows et al., 1982) was assessed by trained evaluators at the time of CIDR insertion. Cows that were < 40 d postpartum (**DPP**) were omitted from the study. Sires (*n* = 26) and AI technicians (*n* = 12) for the beef cows were all unique to their locations.

Exposure of beef cows to natural service sires occurred no sooner than 10 d post-FTAI. Natural service sire exposure continued through the remainder of each location’s breeding season. Pregnancy outcomes were assessed twice via transrectal ultrasonography. The first assessment was conducted 30 to 45 d post-FTAI, the second assessment was conducted 30 d after the end of each location’s breeding season.

### Management of dairy cows

Lactating dairy cows (*n* = 2,208) managed in one organic-certified herd in CO were enrolled and primarily included Holsteins with some Holstein × Jersey crosses.

Dairy cows were housed in sand-bedding-free stalls with free access to an adjacent dry lot. After calving, all dairy cows were milked twice daily with an average milk yield of 30 kg/cow/d. Dairy cows were fed a mixed ration twice daily to meet or exceed nutritional requirements for a lactating Holstein cow producing 30 kg/d of milk with 3.5% fat and 3.1% true protein (NRC, 2001). During the grazing season (April to September), dairy cows had access to pasture. Grazing provided at least 30% of the DMI of the total ration. The ration was based on corn silage (5% to 7%), wheat silage (17% to 19%), grain mix containing soybean, soy hulls, corn, wheat, and minerals and vitamins (38% to 41%), sorghum silage (5% to 7%), alfalfa hay (2%), grass hay (0% to 1.5%), and pasture grazing (estimated as 30% to 38%).

No artificial hormones were used to manage reproduction and dairy cows received AI based on visual estrous detection. Estrus detection was conducted by trained evaluators who also acted as the primary inseminators. Each cow’s tailhead was chalked daily with an oil-based marking chalk. Estrus was determined to have occurred due to an observed lack of chalk on the tailhead in conjunction with physical signs of estrus being displayed by the cow. Body condition score (1 = severe under condition; 5 = severe over condition; Wildman et al., 1982) was assigned to each cow at the time of AI. Cows that were < 40 d in milk (**DIM**) at the time of AI were omitted from the study. Insemination involved multiple sires (*n* = 26) and AI technicians (*n* = 6). Pregnancy outcomes were assessed by transrectal ultrasonography 32 ± 3 d post AI, with a positive confirmation of pregnancy due to the presence of a live embryo with a heartbeat. Ultrasound rechecks occurred 60 ± 3 d post AI to confirm maintenance of pregnancy. Cows that received AI due to a subsequent observation of estrus were considered not pregnant. The mean ± SD number of inseminations per dairy cow was 2.4 ± 1.5.

### Statistical analyses

Binomial variables (pregnant, not pregnant) were analyzed utilizing logistic regression via the GLIMMIX procedure in SAS (SAS Inst. Inc., Cary, NC). Two separate models were constructed to analyze the pregnancy risk (PR) to AI and season-long PR within beef cows. A third model was constructed to analyze the PR of AI for dairy cows. Models used to analyze PR to AI and season-long PR for beef cows included fixed effects of treatment and parity (primiparous vs. multiparous). Random effects included DPP at FTAI (continuous variable), location (ranch), and sire. The model assessing PR to AI in beef cows also included the continuous variable BCS; however, BCS was removed from the model for season-long PR because *P* > 0.1. In determining the risk of pregnancy to AI, the interactions of parity × treatment (*P* < 0.05) and DPP × treatment (*P* = 0.09) remained in the model. No interactions were significant for season-long PR in beef cows. Chi-square via the FREQ procedure of SAS was utilized to determine summary statistics by location.

The model analyzing PR to AI for dairy cows included the fixed effects of treatment, technician, and stage of lactation at AI (40 to 80 DIM, 81 to 130 DIM, or 131 to 380 DIM). Average daily production of milk was included as a continuous random variable. Sire was included as a random effect in the model. The fixed effect of pen and the continuous variables of BCS, lactation, and time inseminated were all considered in the model utilizing backward stepwise selection but ultimately were removed if *P* > 0.1. Stage of lactation at AI × treatment (*P = *0.09) was the only interaction that remained in the model. Chi-square via the FREQ procedure in SAS was utilized to determine summary statistics by pen. Differences with *P* ≤ 0.05 were considered significant and *P* ≤ 0.1 were considered tendencies.

## Results

### Supplementation of TGF at AI in beef cows

The overall risk of pregnancy to AI for all beef cows was not affected (*P *= 0.274) by TGF treatment (CON = 58.1% vs. TGF = 53.1%; Table 1). The risk of pregnancy to AI for primiparous beef cows was not affected (*P *= 0.098) by TGF treatment (CON = 62.5% vs. TGF = 48.2%; Table 2), and season-long PR in primiparous females was not affected by treatment (*P *= 0.405). The risk of pregnancy to AI for multiparous beef cows was not affected (*P *= 0.203) by TGF treatment (CON = 53.6% vs. TGF = 57.9%; Table 1), and season-long PR in multiparous females was not affected by treatment (*P *= 0.801); however, DPP at FTAI (*P *< 0.025) and parity (*P *< 0.038) both affected season-long risk pregnancy in multiparous cows. Location B was an outlier for PR to AI (52.5%), and season-long PR (83.2%) compared to the mean PR to AI (55.9%), and season-long PR for all other locations (92.2%).

**Table 1.** Pregnancy risk to AI and season-long pregnancy risk assessed at the end of the breeding season in lactating beef cows by parity and combined^1^

		Treatment^2^		*P*-value^2^
Parity	Variable	CON	TGF	
Primiparous^5^	Number	86	84	—
	PR to AI^3^, %	62.5	48.2	0.098
	Season-long PR^3^^,^^4^, %	90.8	86.1	0.405
Multiparous^5^	Number	471	489	—
	PR to AI^3^, %	53.6	57.9	0.203
	Season-long PR^3^^,^^4^, %	93.3	93.7	0.810
Combined	Number	559	573	—
	PR to AI^3^, %	58.1	53.1	0.274
	Season-long PR^3^^,^^4^, %	91.5	91.2	0.881

^1^All beef cows were subjected to a 7 d CO-Synch + CIDR estrous synchronization protocol and evaluated for pregnancy risk (**PR**) to AI and season-long pregnancy risk.

^2^Cows were randomly assigned at fixed-time AI to either receive control (**CON**) semen or semen treated with 10 ng of recombinant human transforming growth factor beta-1 (**TGF**) post-thaw.

^3^LS means.

^4^Eight cows were not present for season-long pregnancy diagnosis but were present for diagnosis of pregnancy risk to AI.

^5^Two cows were excluded due to unknown parity status.

**Table 2.** Summary statistics for the overall study population of beef cows by location and herd^1^

Location	Herd	No.	Primiparous, %	Days postpartum at AI^2^	BCS^2^^,^^3^	Pregnancy risk to AI^4^, %	Season-long pregnancy risk^5^, %
A	1	185	0.0	73 ± 10	5.5 ± 0.5	56.0	92.3
B	2	45	13.3	76 ± 15	5.3 ± 0.5	51.8	82.4
B	3	43	11.6	74 ± 14	5.1 ± 0.6	53.1	84.0
C	4	68	20.6	80 ± 13	5.2 ± 0.5	49.9	91.5
C	5	55	23.6	77 ± 15	4.9 ± 0.6	58.6	90.0
D	6	63	0.0	93 ± 11	6.3 ± 0.6	60.3	95.3
D	7	93	23.7	67 ± 15	6.1 ± 0.8	53.3	92.4
E	8	98	4.12	57 ± 8	5.5 ± 0.4	58.8	91.5
E	9	128	28.9	85 ± 7	5.4 ± 0.4	52.3	90.0
F	10	196	23.6	92 ± 10	4.9 ± 0.6	57.1	95.4
F	11	158	14.6	70 ± 14	5.0 ± 0.6	56.3	91.0
Overall		1,132	15.0	77 ± 12	5.4 ± 0.6	55.2	90.5

^1^Lactating beef cows (*n* = 1,132) in 11 herds (i.e., subset of cows) at six locations (ranches) across two states were enrolled.

^2^Mean ± SD.

^3^BCS (1 = thin, 9 = obese).

^4^Assessed at 30 to 45 d after AI by transrectal ultrasonography.

^5^Assessed at a minimum of 30 d post completion of the breeding season by transrectal ultrasonography, adjusted mean percentages.

**Figure 1.**
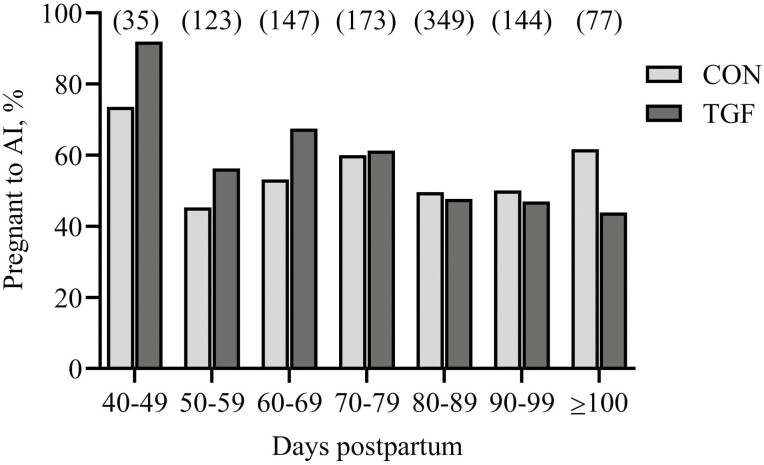
Overall risk of pregnancy to AI in lactating beef cows stratified by days postpartum and treatment. Beef cows (*n* = 1,132) were subjected to a 7 d CO-Synch + CIDR estrous synchronization protocol. Cows were bred by AI following estrus detection or GnRH with control (**CON**) semen alone or semen treated with 10 ng of recombinant human transforming growth factor beta-1 (**TGF**). Data represents the proportion of cows that were pregnant following AI according to treatment and the days postpartum that AI was completed. Numbers of cows in each period are indicated in parentheses. There was a tendency (*P* = 0.089) for a DPP × treatment interaction; however, mean pregnancy risk to AI did not differ at any of the days postpartum when AI was completed (*P* > 0.10).

Risk of pregnancy to AI in beef cows, stratified by DPP, is included in Figure 1. Mean PR to AI did not differ between treatments during any of the time periods (*P* > 0.10). There was a tendency (*P *< 0.085) for a DPP × treatment interaction for PR to AI. Among cows inseminated at 40 to 69 DPP, those receiving TGF had a numerical increase (*P* > 0.05) in PR to AI. Conversely, CON cows were numerically higher (*P* > 0.05) for PR to AI compared to TGF among those inseminated ≥ 100 DPP.

Summary statistics for beef cows by location (ranch) and herd (a subset of cows managed as a group within a location) are reported in Table 2. The number of primiparous beef cows enrolled in the study was 170 (15.0%). Herds 1 and 6 were both composed entirely of multiparous females due to retaining a large number of females the prior year and(or) having primiparous females at a separate location in a separate herd. Herds 6 and 10 had the longest numerical DPP at the time of FTAI, while herd 8 had the shortest. Cows in location D had the highest numerical BCS, while the other locations were consistent with each other.

For beef cows in this experiment, BCS was a significant (*P *< 0.008) factor affecting the risk of pregnancy to AI. However, there is no clear indication of a specific location or herd functioning as an outlier (Table 2). Locations D and F had the highest numerical PR to AI (56.8% and 56.7%, respectively) and season-long PR (93.9% and 93.2%, respectively) while also having a difference of more than one BCS between the two locations (6.2 and 4.9). For the risk of pregnancy to AI, the parity × treatment interaction was significant (*P *< 0.047), with TGF resulting in a numerical decrease in primiparous cows and a numerical increase in multiparous cows (Table 1).

### Supplementation of TGF at AI in dairy cows

**Figure 2.**
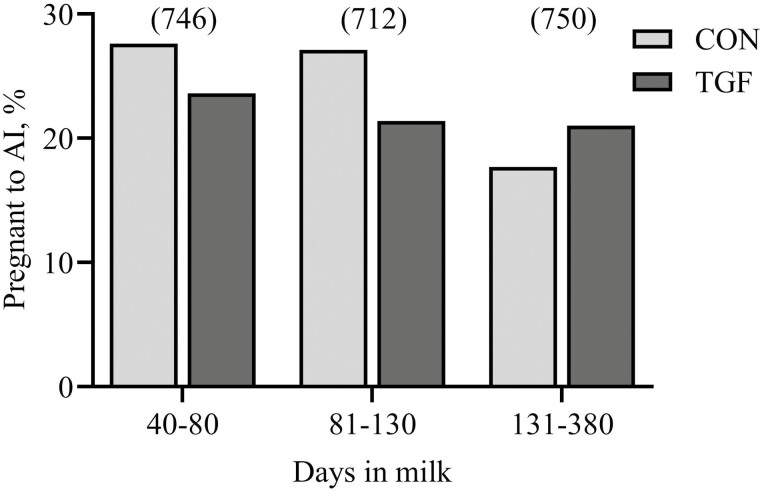
Overall risk of pregnancy to AI in organic dairy cows by days in milk and treatment. Dairy cows (*n* = 2,208) received AI after natural expression of estrus. Cows were randomly assigned at AI to receive either control (**CON**) semen alone or semen treated with 10 ng of recombinant human transforming growth factor beta-1 (**TGF**). Data represents the proportion of cows that were pregnant following AI according to treatment and the period of lactation that AI was completed (40 to 80 DIM, 81 to 130 DIM, 131 to 380 DIM). Numbers of cows in each period are indicated in parentheses. There was a tendency (*P* = 0.091) for a DIM × treatment interaction; however, mean pregnancy risk to AI did not differ between treatments for any of the DIM categories (*P* > 0.10).

For dairy cows, there was no difference between CON and TGF for risk of pregnancy to AI overall (*P* = 0.317), among primiparous females (25.3 vs. 35.1%, respectively; *P *= 0.209), or among multiparous females (23.9 vs. 21.1%, respectively; *P *= 0.141). Stage of lactation at AI (*P *= 0.012) and average daily milk yield (*P *= 0.001) affected the risk of pregnancy to AI. Although only 6 trained individuals performed the inseminations, there was a tendency for technicians to effect (*P *= 0.082) risk of pregnancy to AI. The tendency (*P *= 0.091) for a DIM × treatment interaction resulted in CON cows in both the early (40 to 80 DIM) and middle (81 to 130 DIM) stages of lactation having numerically greater PR to AI than TGF, while in the late (131 to 380) stage of lactation PR to AI was numerically greater for TGF than CON cows (Figure 2).

Summary statistics for the dairy cows are reported by pen in Table 3. The number of primiparous cows enrolled in the study was 145 (6.8%), with pen nine containing the most (numerically and as a percentage). The overall range for DIM at AI among individual cows was 40 to 380 d. Pen one had the shortest numerical DIM at AI and > 3.0 mean lactations, but still had the second highest numerical risk of pregnancy to AI (25.5%). Generally, there was limited variation among pens for BCS, mean lactation number, and PR. It is worth noting that the two pens (two and eight) with the highest numerical number of inseminations also had the highest numerical DIM.

**Table 3.** Summary statistics for the overall study population of dairy cows by pen^1^

Pen	No.	Primiparous, %	Days in milk at AI^2^	BCS^2^^,^^3^	Lactation^2^	Number of AI^2^	Pregnancy risk to AI^4^, %
1	240	2.5	98 ± 48	2.9 ± 0.4	3.2 ± 1.4	2.0 ± 1.3	25.5
2	220	4.1	130 ± 69	3.0 ± 0.4	2.9 ± 1.2	2.8 ± 2.0	25.4
3	276	4.7	112 ± 54	3.0 ± 0.4	3.1 ± 1.3	2.3 ± 1.5	21.9
4	267	5.2	110 ± 51	2.9 ± 0.3	2.8 ± 1.2	2.3 ± 1.4	22.8
5	293	6.8	111 ± 56	2.9 ± 0.3	2.9 ± 1.3	2.4 ± 1.5	27.8
6	268	7.5	111 ± 51	2.9 ± 0.3	2.9 ± 1.3	2.3 ± 1.4	22.6
7	235	9.4	127 ± 55	2.9 ± 0.4	3.0 ± 1.3	2.5 ± 1.5	22.9
8	232	6.0	135 ± 56	2.9 ± 0.3	2.9 ± 1.3	2.8 ± 1.6	17.7
9	177	15.3	109 ± 60	2.9 ± 0.3	2.8 ± 1.5	2.1 ± 1.5	21.3
Overall	2,208	6.8	116 ± 56	2.9 ± 0.3	2.9 ± 1.3	2.4 ± 1.5	23.1

^1^Organic dairy cows (*n* = 2,208) at one location with nine pens.

^2^Mean ± SD.

^3^BCS (1 = severe under condition, 5 = severe over condition).

^4^LS mean percentages; assessed at 30 to 45 d after AI by transrectal ultrasonography or by the re-insemination of the cow due to a subsequent observation of estrus.

## Discussion

Seminal plasma elicits cellular and molecular changes in the endometrium at insemination that improve pregnancy outcomes in cattle, swine, and mice (Murray et al., 1983; Bromfield et al., 2014; Ortiz et al., 2019). One bioactive molecule in seminal plasma responsible for molecular and cellular responses in the endometrium is TGF. Here we hypothesized that supplementation of semen with TGF at the time of AI would increase PR to AI. The data presented here suggests that supplementation of TGF at the time of AI neither compromises or improves PR to AI in beef or dairy cows.

The fornix vagina is the natural site of semen deposition in cattle. From there, sperm travel through the cervix into the uterus leaving most seminal plasma in the vagina, lost to retrograde transport (Rizo et al., 2019). It is unclear how much, if any, seminal plasma is delivered to the uterus in cattle during natural cover. In the case of AI, semen deposition bypasses the cervix, delivering the extended semen directly into the uterine body (Rizo et al., 2019). The dilution of semen with extenders, the use of washed sperm from X-sorted semen and the use of small insemination doses of semen results in very little if any exposure of maternal tissues to seminal plasma. It has been suggested that sperm may carry seminal plasma components and(or) proteins into the uterus that were acquired during ejaculation or modulate the uterine environment independently of seminal plasma (Schjenken et al., 2021). Mating cows to vasectomized bulls where sperm were absent and seminal plasma was deposited into the vagina did not elicit changes to the endometrial transcriptome compared to cows bred by intact bulls (Recuero et al., 2020).

Under natural conditions, mating occurs at estrus; whereas AI that incorporates the AM:PM rule, deposition of frozen-thawed semen occurs approximately 12 h after the detected onset of estrus (Trimberger, 1948). With ovulation occurring 28 to 30 h after the onset of estrus (White et al., 2002) the female genital tract is exposed to seminal fluids and would likely initiate an inflammatory response before ovulation or fertilization (Odhiambo et al., 2009).

Ibrahim et al. (2019) described the impact of semen and seminal plasma on the bovine endometrium by altering the expression of inflammatory mediators in cultured endometrial cells and explants. Conversely, the authors reported that 24 h after intrauterine infusion of seminal plasma, alterations in the endometrium were significantly less than those observed in vitro. There are many species of which pregnancy can be achieved in the absence of seminal plasma, utilizing in vitro fertilization or AI via washed sperm (Lima et al., 2009; Ibrahim et al., 2019). While much of the modern dairy industry relies heavily on AI and the use of high genetic merit sires, the beef industry still relies heavily on the use of natural cover (Bromfield, 2016). In the United States, over 10 million bovine inseminations are performed utilizing semen where seminal plasma is considerably diluted during semen extension to maximize the efficiency of a single ejaculate (Vishwanath, 2003). Studies have demonstrated that pregnancy rates in cows that underwent AI or embryo transfer, in the absence of seminal plasma, were at least equal to natural conception (Sartori et al., 2006; Lima et al., 2009).

Odhiambo et al. (2009) evaluated the supplementation of seminal plasma, TGF, or bovine serum albumin at the time of AI in multiparous beef cows over 3 years of trials. The authors found that pregnancy outcomes were similar among treatments but differed between years (*P *< 0.050). Overall conception rates were 54.7% ± 5.3%, 54.8% ± 5.9%, and 53.1% ± 5.3% for seminal plasma, TGF, and bovine serum albumin, respectively. The authors also observed a gradual decrease in the overall conception rate over time (year 1 = 69.8% ± 6.7%; year 2 = 52.5% ± 5.3%; year 3 = 40.3% ± 4.6%). In year 3 TGF increased the overall conception rate when fertility was compromised (seminal plasma, 33.1% ± 7.9%; TGF, 49.2% ± 7.9%; bovine serum albumin, 38.4% ± 7.9%; Odhiambo et al., 2009). Combined these studies suggest that seminal plasma is not required to achieve pregnancy in cattle or other species; however, the question of optimizing pregnancy outcomes like birth weight or postnatal phenotype using seminal plasma is yet to be fully resolved.

Ortiz et al. (2019) conducted an experiment using dairy cows similar to the current study, in which an additional 0.5 mL straw of pooled seminal plasma or saline was applied intrauterine at the time of insemination in first-service lactating Holstein cows. The authors used 511 primiparous and 554 multiparous females that were inseminated with X-sorted sperm, while an additional 627 multiparous cows were inseminated with conventional semen (Ortiz et al., 2019). The cows were synchronized using a Double Ovsynch protocol, which is different than the current study in which cows were detected for a natural estrus (vs. a synchronized estrus). Ortiz et al. (2019) reported no effect of seminal plasma infusion on the risk of pregnancy to AI in cows at 32 or 60 d post AI or on pregnancy loss. For the conventional semen, the authors reported that the inclusion of seminal plasma reduced the risk of pregnancy to AI at 32 d post AI (45.5 vs. 52.4 %), while seminal plasma had no effect when X-sorted semen was used. In the current study, we observed a numerical difference in risk of pregnancy to AI in favor of CON (9.8 percentage points) over TGF among primiparous females, but it was not statistically significant. Interestingly, Ortiz et al. (2019) demonstrated a tendency (*P* = 0.097) for seminal plasma infusion to increase heifer birth weight compared to controls, and when considering heifers conceived with X-sorted semen only, birth weight was significantly increased when conceived in the presence of seminal plasma (*P* = 0.019). These data are similar to those observed in mice, where offspring conceived by sires in which the seminal vesicles were surgically removed grow heavier into adulthood due to increased adiposity (Bromfield et al., 2014). In addition, the metabolic phenotype of these rodent offspring is altered resulting in hypertension, disrupted metabolic hormone profiles, and reduced glucose tolerance (Bromfield et al., 2014). It is unclear what the adult metabolic phenotype of cattle conceived in the presence of seminal plasma or TGF will be.

Odhiambo et al. (2009) found that intrauterine infusion of TGF at the time of AI increased pregnancy rates in cattle with low fertility; however, similar results were not observed in the current study. Here, dairy cow fertility that was compromised via reproductive issues of unknown origins, or that took longer to get pregnant by AI, did not benefit from supplementation of TGF (although their PR to AI was numerically higher). Beef cows inseminated at fewer DPP had numerically higher PR to AI but did not benefit from the use of TGF at AI.

In dairy cattle, the highest incidence of pregnancy loss occurs within the first week of pregnancy when approximately 40% of embryos fail to develop beyond the blastocyst stage (Santos et al., 2004; Wiltbank et al, 2016). This window of development is a critical point for modulation of the maternal environment which could increase the pregnancy success of a cow and may be modulated by seminal plasma or TGF (Ibrahim et al., 2019; Rizo et al., 2019). However, the lack of a positive effect on fertility using seminal plasma reported by Ortiz et al. (2019) and Odhiambo et al. (2009) or TGF reported here or by Odhiambo et al. (2009), raises questions about the biological significance of intrauterine supplementation with semen components in cattle. If we had detected a positive effect of TGF supplementation on PR here, we would likely require an additional, large field trial whereby TGF was loaded directly into semen straws at packaging to correct for the addition of BSA and gentamycin to the TGF stock solution used in our treatment. Timing differences in the rate of development following insemination in the cow versus the mouse may make postcoital inflammation important in one species but not the other. Ultimately, there was no effect on the risk of pregnancy following supplementation of semen with TGF in beef or dairy cows. The impact of such an intervention on postnatal phenotype will require further investigation.

## Abbreviations

CIDR: controlled internal drug release
DIM: days in milk
DPP: days postpartum
FTAI: fixed-time artificial insemination
GnRH: gonadotropin-releasing hormone
PGF: prostaglandin F_2α_
PR: pregnancy risk
TGF: transforming growth factor beta-1
